# Bronchial Dieulafoy's Disease in Children: A Case Report and Review of Literature

**DOI:** 10.3389/fped.2020.00273

**Published:** 2020-05-28

**Authors:** Yi-Ting Yeh, Madhavan Ramaswamy, Jinnie Shin, Denise McIntyre, Neil McIntosh, Jean-Nicolas Racicot, Samantha Chippington, Samuel Stuart, Nagarajan Muthialu

**Affiliations:** ^1^Department of Cardiothoracic Surgery, Great Ormond St Hospital, London, United Kingdom; ^2^School of Medicine, National Yang-Ming University, Taipei, Taiwan; ^3^Department of Interventional Radiology, Great Ormond St Hospital, London, United Kingdom

**Keywords:** bronchial, vascular malformation, surgery, sleeve resection of bronchus, haemoptysis

## Abstract

Dieulafoy's disease is a rare vascular lesion characterized by presence of large aberrant arteries within the submucosa of gastrointestinal tract or respiratory tract with a potential to cause life-threatening hemorrhage. Treatment includes bronchoscopy ablation, angiographic embolization or surgery. We report management of 7-year old girl with Dieulafoy's disease in the airway who presented with recurrent hemoptysis. Bronchial angiography revealed multiple feeding vessels to the lesion. Considering the potential risk of recurrence with embolization, sleeve resection of bronchus offered complete resolution. This case demonstrates the usefulness of bronchial angiography as part of multi-faceted approach before surgery in the management of Dieulafoy's disease.

## Introduction

Dieulafoy's disease (DD) is a distinct pathologic condition characterized by presence of large aberrant arteries within the submucosa of the gastrointestinal tract (1). Most commonly present in the stomach with a potential to cause life-threatening hemorrhage, its occurrence in airway is rare (2, 3).

## Case Report

Our patient was a 7-year old girl who presented with frequent episodes of unprovoked massive hemoptysis for 5 months. She was referred to us after a bronchoscopic finding of a polypoid lesion in her bronchus intermedius (RBI). On admission to our hospital, she appeared well but continued to have small-volume hemoptysis. Physical examination was essentially normal, especially with no cutaneous telangiectasia.

**Figure 1:**
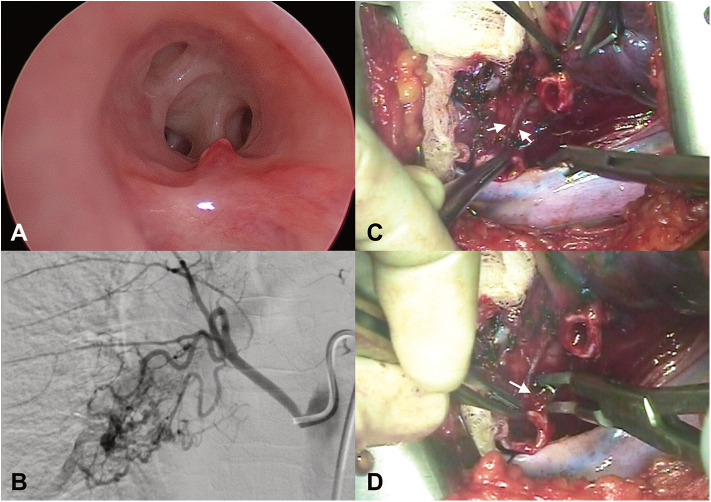
**(A)** Bronchoscopy showed a polypoid lesion over the right bronchus intermedius. **(B)** Bronchial artery angiography showed multiple engorged and tortuous feeding vessels to the lesion. **(C,D)** Intraoperative photographs showing discrete engorged feeding arteries (arrows) on the wall of the bronchus intermedius.

Computer tomography angiography (CTA) and a repeat bronchoscopy revealed an abnormal vascular lesion in her RBI (Figure 1). To further characterize the lesion, angiography of the bronchial artery was performed, It showed multiple tortuous enlarged feeding vessels (Figure 1). Considering the non-negligible risk of recurrence and the fact that the patient was referred from overseas, a sleeve resection of the RBI was performed over angiographic embolization after a multidisciplinary discussion. She had an uneventful post-operative recovery.

Histopathology revealed increased number of thick-walled vessels with prominent intimal thickening within submucosa and peri-bronchial connective tissue of the resected specimen. Places of erosion of the respiratory epithelium close to these thick-walled vessels could be found. Patchy chronic inflammation in the submucosa and patchy hypertrophy of the sub-epithelial connective tissue were also present (Figure 1). Currently, she remains asymptomatic and there is no evidence of recurrence of the lesion nor any stenosis at the site of anastomosis in the follow-up bronchoscopy performed 6 months after the surgery.

## Discussion

Dieulafoy's disease of the bronchus was first described in 1995 by Sweerts et al. (2) and to date fewer than 60 cases have been reported. The pathology is characterized by wide submucosal branches of bronchial artery with intraluminal protrusion and a tendency for erosion or spontaneous rupture resulting in massive haemoptysis (2, 3).

Its pathogenesis is hypothesized to be a hypertrophic response to chronic inflammation in a background of a possible congenital malformation. Patients in reported series are mostly middle-aged individuals with a male preponderance and with a history of smoking (3). Pediatric cases are exceedingly rare and to the best of our knowledge, only four cases have been reported in the literature (4–6).

Recurrent massive hemoptysis was the most common cause of hospitalization of those children described in the literature. Bronchoscopy in all these patients showed polypoid and non-pulsatile lesions in their airways. A white cap was described in one patient. The vascular lesions were able to be identified by CTA in three cases. It was non-diagnostic in the fourth patient, in whom an endobronchial ultrasound revealed the underlying lesion.

One patient underwent upfront segmentectomy due to lack of expertise in interventional radiology in their facility. Rest of the patients (*n* = 2) underwent bronchial artery embolization (BAE) with initial success in one of them (50%). Lobectomy was performed in one patient after failure of BAE (50%). Recurrence after initial treatment was reported in two patients (66%, 30 and 52 mo post-procedure, respectively) of which one underwent lobectomy and the other, continued with conservative management. Histological examination of all those published cases showed dilated tortuous branches of bronchial artery in the submucosa of the respiratory tract in association with mucosal erosion.

Based on our experience of managing difficult hemoptysis in children, accurate localization of the exsanguinating lesion and the extent of the disease of our case were achieved by diagnostic angiography along with CTA and bronchoscopy (7). Intra-operative endobronchial ultrasound may have been useful to delineate the extension of submucosal vessels but was not utilized in our case due to lack of expertise (5).

The optimal management of these lesions in children remains controversial. BAE, with its relative non-invasiveness as compared to surgery, may be considered as a first-line treatment or for emergent haemostasis (7). There is, however, little evidence of its use in children. The recurrence rate in the reported pediatric patients is high (see Table 1: two of the three reported cases, 66%) when compared to adult patients. In a recent review, among the 30 adult patients with DD of the bronchus, three patients required surgery for recurrence after BAE and eight required surgery due to unsuccessful BAE (3). There were reports of recurrences well after a year of BAE suggesting that some part of the lesion might have been overlooked during initial treatment or shunting after embolization would have resulted in its recurrence (3, 6). Presence of multiple feeding vessels in our case proved to be challenging for complete embolization with high risk of recurrence.

**Table 1:** Literature review on bronchial Dieulafoy's disease in children.

**Case**	**Age/sex**	**Location**	**Management (outcome)**		**Recurrence (interval)**	**Management for recurrence**	**References**
			**BAE**	**Operation**			
1	13 y/male	RLL	Success	–	Yes (3 mo)	Thoracotomy with bilobectomy	(4)
2	16 y/female	RLL	–	Right basal segmentectomy (success)	No	n/a	(5)
3	8 m/female	RUL	Failure	RUL lobectomy (success)	Yes (52 mo)	Conservative	(6)
4 (current)	7 y/female	RIMB	–	Sleeve resection of RIMB (success)	No	n/a	

*BAE, bronchial arterial embolisation; RBIM, right intermediate bronchus; RLL, right lower lobe; RUL, right upper lobe*.

BAE as well as any surgical procedure is not entirely without complications. Spinal cord ischemia, inadvertent embolization of the brain and other non-target embolization have been reported (7).

Our case highlights that CTA and bronchial angiography should be integral parts of investigations for severe hemoptysis in children in addition to a thorough bronchoscopy examination so that accurate diagnosis can be reached. BAE may be considered in the treatment of Dieulafoy's disease in children, but surgical resection using parenchymal preservation technique (sleeve resection) can be more appropriate in selected scenarios where definitive treatment with lower risk of recurrence is preferred.

## Data Availability Statement

The raw data supporting the conclusions of this article will be made available by the authors, without undue reservation, to any qualified researcher.

## Ethics Statement

Written informed consent was obtained for the publication of this case report from the parents of the child treated for such condition while in hospital and they are fully aware of the images as well as the report sent across to the journal.

## Author Contributions

Y-TY and MR prepared the initial draft. SC, DM, and NMc worked on editing the pictures. JS, J-NR, SS, and SC contributed to interventional radiology component. NMu edited the final version and approved for submission.

## Conflict of Interest

The authors declare that the research was conducted in the absence of any commercial or financial relationships that could be construed as a potential conflict of interest.
